# Human Immune Responses to Melioidosis and Cross-Reactivity to Low-Virulence *Burkholderia* Species, Thailand^1^

**DOI:** 10.3201/eid2603.190206

**Published:** 2020-03

**Authors:** Patpong Rongkard, Barbara Kronsteiner, Viriya Hantrakun, Kemajittra Jenjaroen, Manutsanun Sumonwiriya, Panjaporn Chaichana, Suchintana Chumseng, Narisara Chantratita, Vanaporn Wuthiekanun, Helen A. Fletcher, Prapit Teparrukkul, Direk Limmathurotsakul, Nicholas P.J. Day, Susanna J. Dunachie

**Affiliations:** University of Oxford, Oxford, UK (P. Rongkard, B. Kronsteiner, N.P.J. Day, S.J. Dunachie);; Mahidol-Oxford Tropical Medicine Research Unit, Bangkok, Thailand (P. Rongkard, V. Hantrakun, K. Jenjaroen, M. Sumonwiriya, P. Chaichana, S. Chumseng, N. Chantratita, V. Wuthiekanun, D. Limmathurotsakul, N.P.J. Day, S.J. Dunachie);; Mahidol University, Bangkok (N. Chantratita, D. Limmathurotsakul);; London School of Hygiene and Tropical Medicine, London, UK (H.A. Fletcher);; Sunpasitthiprasong Hospital, Ubon Ratchathani, Thailand (P. Teparrukkul)

**Keywords:** Burkholderia pseudomallei, Burkholderia thailandensis, Burkholderia thailandensis CPS variant, melioidosis, capsular polysaccharide, immune cross-reactivity, indirect hemagglutination assay, ELISA, interferon-γ ELISpot, whole blood stimulation assay, Thailand, bacteria

## Abstract

Melioidosis is a neglected tropical disease with an estimated annual mortality rate of 89,000 in 45 countries across tropical regions. The causative agent is *Burkholderia pseudomallei*, a gram-negative soil-dwelling bacterium. In Thailand, *B. pseudomallei* can be found across multiple regions, along with the low-virulence *B. thailandensis* and the recently discovered *B. thailandensis* variant (BTCV), which expresses *B. pseudomallei*–like capsular polysaccharide. Comprehensive studies of human immune responses to *B. thailandensis* variants and cross-reactivity to *B. pseudomallei* are not complete. We evaluated human immune responses to *B. pseudomallei*, *B. thailandensis*, and BTCV in melioidosis patients and healthy persons in *B. pseudomallei–*endemic areas using a range of humoral and cellular immune assays. We found immune cross-reactivity to be strong for both humoral and cellular immunity among *B. pseudomallei*, *B. thailandensis*, and BTCV. Our findings suggest that environmental exposure to low-virulence strains may build cellular immunity to *B. pseudomallei*.

Melioidosis is a neglected tropical disease with an estimated annual global mortality rate of 89,000 (*1*). Its cause is the gram-negative bacterium *Burkholderia pseudomallei*, which is found in environmental soil and water in Southeast Asia and northern Australia and is increasingly recognized across tropical regions (*2*). Known underlying risk factors that contribute to increased susceptibility to infection include diabetes, chronic kidney diseases, and alcohol abuse (*3*). The rapid expansion of type 2 diabetes, especially in low- and middle-income countries, is likely to exacerbate the situation further (*4*). In Thailand, *B. pseudomallei* is highly distributed in the environment in the northeast, where most of the country’s melioidosis cases have been reported (*5*). However, *B. pseudomallei* is also isolated from soil in the eastern and central parts of Thailand. A closely related species of minimal virulence, *B. thailandensis*, and a *B. thailandensis* variant (BTCV) expressing the *B. pseudomallei*–like capsular polysaccharide (CPS) are also present in the soil in Thailand (*6*). The genomic composition of *B. thailandensis* shows >85% similarity with *B. pseudomallei* (*7*). However, there are a few key differences, including the lack of virulence factors, such as capsular polysaccharide, and the presence of the arabinose assimilation operon (*8*) in *B. thailandensis* and BTCV. The hybrid BTCV exhibits several features found in *B. pseudomallei*, including resistance to decomposition by the complement system, intracellular survival inside macrophages, and colony morphology that resembles that of *B. pseudomallei* (*9*). Although BTCV has acquired *B. pseudomallei*–like CPS gene clusters, it has been shown to be nonpathogenic in mouse models (*9*).

Immune cross-protection conferred by *B. thailandensis* variants has been demonstrated in animal models (*10*). In particular, mice immunized with the BTCV isolate E555 showed superior cross-protection to that from the noncapsulated strain against a lethal dose of *B. pseudomallei* challenge, resulting in high CPS-specific IgG levels and decreased bacterial prevalence (*10*). To date, comprehensive immune cross-reactivity to *B. thailandensis* and BTCV has not been studied in humans.

Protective adaptive immunity in human melioidosis is complex, but defense against its intracellular pathogen is likely to require competent cellular immune responses largely mediated by T lymphocytes (*11*,*12*). Our team and others have shown that surviving melioidosis patients have elevated CD4 and CD8 T-cell–mediated interferon γ (IFN-γ) responses to *B. pseudomallei* compared with deceased case-patients (*13*,*14*). Along with key protective immunity conferred by cellular immune responses, humoral immunity against BP infection has been demonstrated to be a component of protection in rodent models (*15*), although antibody levels measured by indirect hemagluttination assay were not significantly associated with survival (p>0.05) in human patients after adjusting for other parameters (*16*). Our study aimed to characterize the relationship between the human *B. pseudomallei*–specific immune response and responses to *B. thailandensis* and BTCV. We evaluated humoral and cellular immune responses to *B. pseudomallei*, *B. thailandensis*, and BTCV in patients with acute melioidosis, in patients with other gram-negative bacterial infections, and in exposed populations in the endemic region with and without diabetes.

## Materials and Methods

### Study Design

We conducted a prospective observational study during 2015–2017 at Sunpasitthiprasong Hospital, Ubon Ratchathani, Thailand, to evaluate human immune responses to *B. pseudomallei*, *B. thailandensis*, and BTCV. We recruited 4 cohorts: inpatients >20 years of age who had culture-confirmed melioidosis (melioidosis cohort; n = 99), patients who had positive cultures for other gram-negative bacterial infections (OGNI cohort; n = 48), patients who attended the hospital’s diabetes outpatient clinic for diabetes mellitus (DM cohort; n = 98), and healthy control participants from the melioidosis-endemic areas who were household contacts of the melioidosis case-patients (HH cohort; n = 96). The number of samples varied between assays due to sample availability (Table 1). We determined 28-day survival status using the hospital death records and by telephone. We collected blood samples during enrollment and processed them as described previously (*13*). We measured humoral and cellular immune responses using indirect hemagglutination assay (IHA), IgM and IgG ELISA, ex vivo IFN-γ enzyme-linked immunospot assay (IFN-γ ELISpot), and a whole-blood stimulation assay (WBA). The ethics committees of the Faculty of Tropical Medicine, Mahidol University (TMEC 12–014); Sunpasitthiprasong Hospital, Ubon Ratchathani (017/2559); and the Oxford Tropical Research Ethics Committee (OXTREC35–15) approved the study protocol.

**Table 1:** Proportions of samples from 4 patient and control cohorts used to evaluate immune responses to melioidosis, Thailand*

Cohort	No. (%) samples by assay		
	IHA or ELISA	IFN-γ ELISpot	WBA
Melioidosis, n = 99	73 (74)	82 (83)	13 (13)
Healthy household contacts, n = 96	35 (36)	93 (97)	8 (8)
Diabetes control, n = 98	54 (55)	95 (97)	NA
Other gram-negative bacterial infections, n = 48	10 (20)	42 (88)	NA

*IFN-γ ELISpot, interferon-γ enzyme-linked immunospot assay; IHA, indirect hemagglutination assay; NA, not available; WBA, whole-blood stimulation assay.

### Antigen Preparation

We prepared antigens in accordance with published methods, unless otherwise stated (*17*,*18*). For IHA, we used crude culture filtrate antigens from pooled isolates with the following specifications: *B. pseudomallei* Thai clinical isolates 199a and 207a (*19*), *B. thailandensis* Thai environmental isolates E264 (ATCC700388) and STBCC006 (*20*), and BTCV Thai environmental E555 and USA clinical isolate CDC3015869. We prepared *B. thailandensis* and BTCV antigens following a traditional IHA antigen preparation as described previously (*17*). For ELISA, we prepared whole-cell heat-killed antigens from *B. pseudomallei* Thai clinical isolate K96243, BT E264, and BTCV E555 as described previously (*18*). For IFN-γ ELISpot and whole-blood stimulation assay, we used a single culture filtrate antigen of each strain, *B. pseudomallei* 199a, *B. thailandensis* E264, and BTCV E555.

### IHA and ELISA

We determined serologic responses to *B. pseudomallei*, *B. thailandensis*, and BTCV by IHA, as well as IgM and IgG ELISA (*18*). We performed IHA as described previously (*17*), with an IHA titer of >1:80 considered positive (*21*).

### IFN-γ ELISpot

We used a commercial IFN-γ ELISpot assay (Mabtech AB, https://www.mabtech.com) to quantify secreted IFN-γ from peripheral blood mononuclear cell (PBMC) in response to *B. pseudomallei*, *B. thailandensis*, and BTCV antigens as described previously (*13*). In brief, we added PBMC at a density of 2 × 10^5^ cells per well to each of 2 antibody-coated plates and incubated them either in media only or in the presence of antigen for 18–20 h at 37°C: final concentration for *B. pseudomallei* was 88 µg/mL; for *B. thailandensis*, 78 µg/mL; for BTCV, 83 µg/mL; and for purified protein derivative (PPD), 20 µg/mL. We added 1 µg/mL of detector antibody and incubated for 3 h at room temperature, then for 2 h with streptavidin-conjugated ALP at room temperature. We used the AP Conjugate Substrate Kit (Bio-Rad, https://www.bio-rad.com) to develop spots for up to 20 min. We analyzed plates on the CTL ELISpot Reader (CTL Analyzers, http://www.immunospot.com) using the proprietary Smartcount automated settings. Results were reported as spot-forming units (SFU) per million PBMC. Background (PBMC in media only) responses in unstimulated control wells were typically <20 spots and were subtracted from those measured in antigen-stimulated wells. We used phytohemagglutinin (PHA) at a concentration of 10 μg/mL as a positive control.

### WBA

For antigen stimulation, we added 500 μL heparinized blood to a 5 mL polystyrene round-bottom tube (Corning, https://www.corning.com) containing a final concentration of 1 μL/mL anti-human CD28 and CD49d (Becton Dickinson, https://www.bd.com), along with *B. pseudomallei*, *B. thailandensis*, or BTCV antigens, all at a final concentration of 1 mg/mL. We used staphylococcal enterotoxin B (SEB) at a final concentration of 10 µg/mL as positive control and RPMI medium supplemented with 10% fetal bovine serum (FBS) (R10 medium) as negative control. We incubated the tubes at 37°C, 5% CO_2_, 95% humidity. After 18 h, we added a final concentration of 10 μL/mL Brefeldin A (eBioscience, https://www.thermofisher.com) and incubated the assay for another 4–5 h under the same conditions. We then incubated samples with Live/Dead (LD) Fixable Near-IR Dead Cell Stain (Life Technologies, https://www.thermofisher.com) according to the manufacturer’s instructions, followed by a single 10-min incubation with FACS Lysing solution at room temperature for red cell removal (Becton Dickinson). We cryopreserved lysed blood cells in FBS with 10% dimethylsulfoxide (DMSO) (Sigma Aldrich, https://www.sigmaaldrich.com) and stored at −80°C until flow cytometry staining.

### Flow Cytometry

We thawed frozen cells at 37°C and washed them twice with R10 medium, then fixed for 20 min followed by 5 min of permeabilization using BD Cytofix/Cytoperm kit (Becton Dickinson). Then, we stained samples with fluorescently labeled human antibodies for 20 min on ice in the dark: CD3-PerCP (clone: UCHT1; BioLegend, https://www.biolegend.com), CD4-V450 (clone: L200, Becton Dickinson), CD8-BV510 (clone: RPA-T8; BioLegend), CD56-VioBrightFITC (clone: AF12–7H3; Miltenyi Biotec, https://www.miltenyibiotec.com), and IFN-γ-PE (clone: 4S.B3; BioLegend). We then analyzed using a MACSQuant Analyzer 10 (Miltenyi Biotec) and performed flow cytometry analysis using FlowJo software version 10.2 on Mac OS X (Becton Dickinson, https://www.flowjo.com).

### Statistical Analysis

The outcomes of interest were correlation between level of immune responses (measured by IHA titers, optical density of IgM and IgG by ELISA and levels of cytokine-producing cells, and IFN-γ SFU) against 3 antigens: *B. pseudomallei*, *B. thailandensis*, and BTCV. We used Spearman’s correlation coefficient (ρ) to determine the correlation between the levels of immune responses against the 3 antigens. We compared ordinal and continuous variables using a nonparametric Mann-Whitney test (comparing 2 independent groups) and Wilcoxon matched-pairs signed rank test (comparing multiple tests on matched cases). We performed all statistical tests using GraphPad Prism version 7.0b for Mac OS X (GraphPad Software, https://www.graphpad.com).

## Results

### Participants

We enrolled a total of 100 patients with culture-confirmed melioidosis during April 2016–November 2017 into the melioidosis cohort. We excluded 1 patient because of a positive concurrent diagnosis of tuberculosis. We recruited participants at a median of 5 days after admission (interquartile range [IQR] 4–6 days) (Table 2). Diabetes was an underlying condition for two thirds (67%) of the melioidosis cohort. The overall 28-day mortality rate was 30% (30/99), with no significant difference (p = 0.9) for patients with and without diabetes.

**Table 2:** Characteristics of patients and controls in study of immune responses to melioidosis and cross-reactivity to low-virulence *Burkholderia* species, by cohort, Thailand*

Baseline characteristics	Cohort			
	Melioidosis, n = 99	Healthy controls, n = 96	Diabetes controls, n = 98*	Other gram-negative bacterial infections, n = 48
Sex				
M	63 (64)	27 (28)	25 (26)	27 (56)
F	36 (36)	69 (72)	73 (74)	21 (44)
Age, y, median (range)	55 (20–84)	48 (25–69)	53 (41–60)	64 (24–95)
Diabetes†	66 (67)	NA	98 (100)	NA
Died‡	30 (30)	NA	NA	NA
Survived	69 (70)	NA	NA	NA

*Values are no. (%) except as indicated. †Includes patients who were previously diagnosed with diabetes or who have a hemoglobin A1C level >6.5% at time of recruitment. ‡Died within 28 days of study enrollment.

### Humoral Immune Responses by IHA against *B. pseudomallei*, *B. thailandensis*, and BTCV

**Figure 1:**
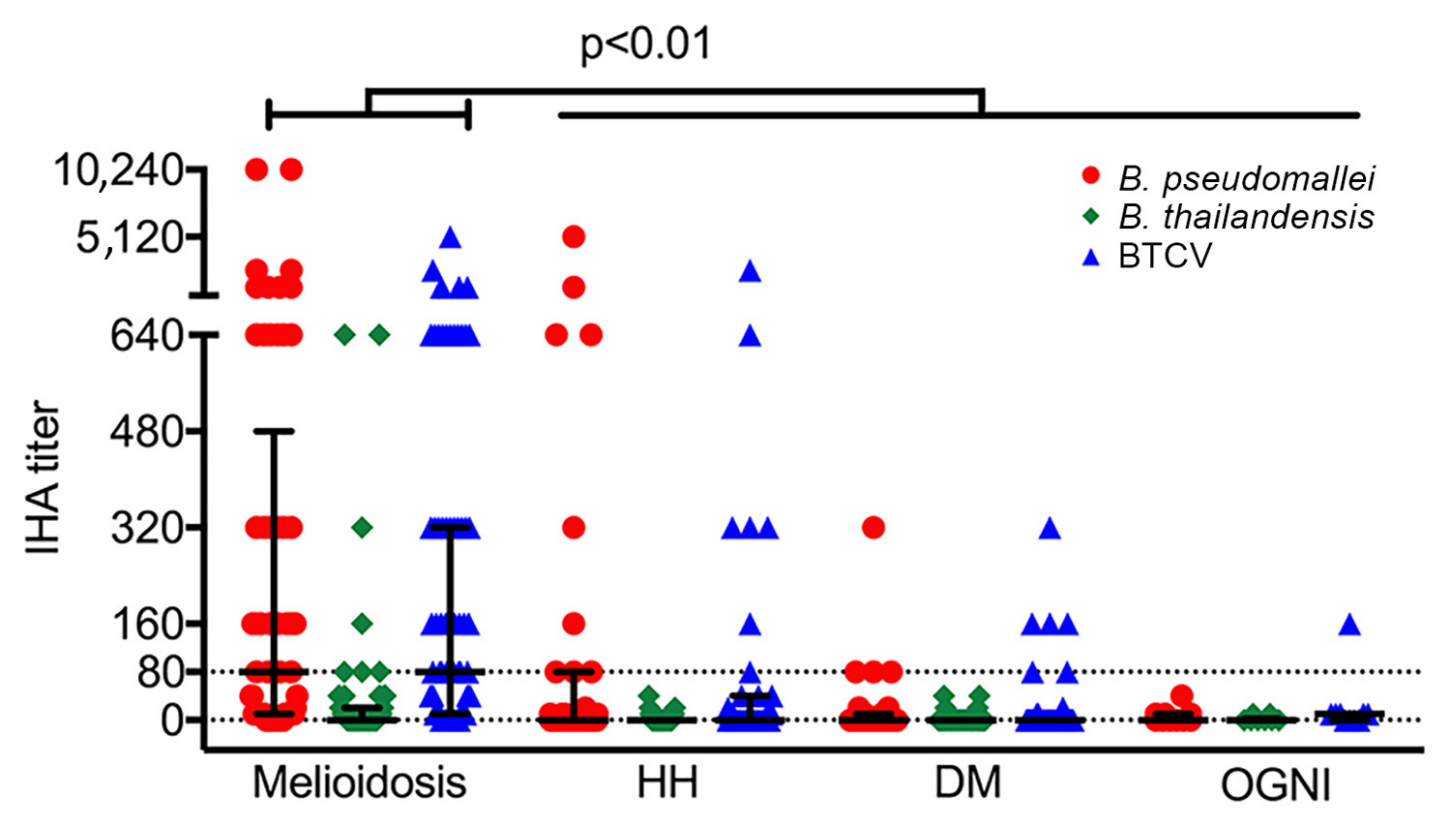
Humoral immune responses to *Burkholderia pseudomallei*, *B. thailandensis*, and BTCV by indirect hemagglutination assay, Thailand. IHA titers are shown for acute melioidosis patients (n = 73) and 3 control cohorts, HH (n = 35), DM (n = 54), and OGNI (n = 10), against culture-filtrate antigen of *B. pseudomallei*, *B. thailandensis*, and BTCV. Each symbol represents an IHA titer response from a patient. Dotted line indicates the IHA cutoff titer for seropositivity. Medians (horizontal lines) and interquartile ranges (error bars) are provided. p values were calculated by using the nonparametric Mann-Whitney test. Horizontal bars at top of figure indicate comparisons across cohorts. BTCV, *B. thailandensis* CPS variant; CPS, capsular polysaccharide; DM, patients with diabetes mellitus; HH, healthy household contacts of the melioidosis case-patients; IHA, indirect hemagglutination assay.

We observed seropositivity against *B. pseudomallei* in 58% (42/73) of the melioidosis cohort, 26% (9/35) of HH, 7% (4/54) of DM, and none (0/10) of OGNI. The median IHA titer against *B. pseudomallei* in the melioidosis cohort (median 1:80, interquartile range [IQR] 1:10–1:320) was higher than that from all control cohorts (HH, median ≤1:10, IQR ≤1:10–1:80; DM and OGNI, median ≤1:10, IQR ≤1:10–1:10; p<0.01). We observed detectable IHA titer against BTCV only in the melioidosis cohort (median 1:80, IQR 1:10–1:320); this result was significantly higher than that for all control cohorts (p<0.01) (Figure 1). We observed strong correlation between *B. pseudomallei* and BTCV IHA in the melioidosis cohort (ρ = 0.96; p<0.01) and the HH cohort (ρ = 0.84; p<0.01). We observed moderate correlation between *B. pseudomallei* and *B. thailandensis* IHA (ρ = 0.53; p<0.01) only in the melioidosis cohort (Appendix Table 1). We detected no responses against *B. thailandensis* in any of the other cohorts (median <1:10).

### Humoral Immune Responses by IgG and IgM ELISA against *B. pseudomallei*, *B. thailandensis*, and BTCV

**Figure 2:**
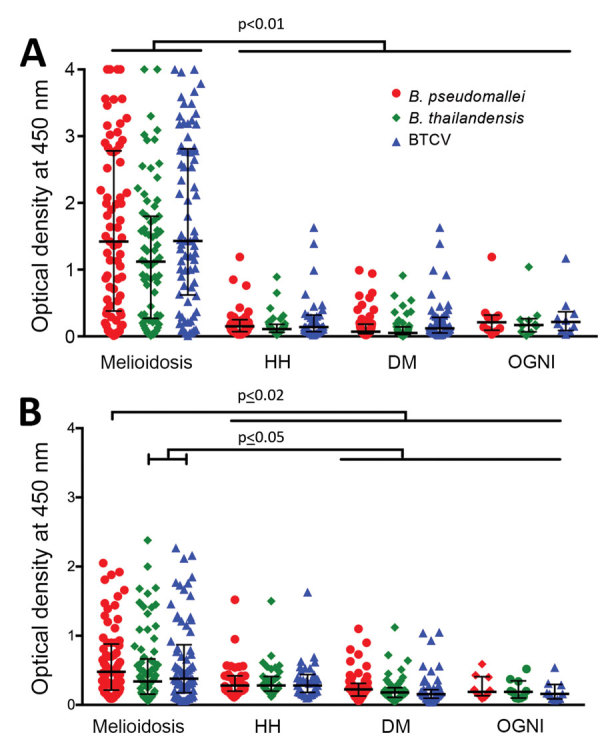
Human humoral immune responses to *Burkholderia pseudomallei*, *B. thailandensis*, and BCTV by IgG and IgM ELISAs, Thailand. IgG-specific (A) and IgM-specific (B) responses are shown for acute melioidosis patients (n = 73) and 3 control cohorts, HH (n = 35), DM (n = 54), and OGNI (n = 10), against culture-filtrate antigen of *B. pseudomallei*, *B. thailandensis* , and BTCV. Each symbol represents an IgM or IgG antibody response from a patient. Medians (horizontal lines) and interquartile ranges (error bars) are shown. p values were calculated by using the nonparametric Mann-Whitney test. Horizontal bars at top of figure indicate comparisons across cohorts. BTCV, *B. thailandensis* CPS variant; CPS, capsular polysaccharide; DM, patients with diabetes mellitus; HH, healthy household contacts of melioidosis case-patients.

The melioidosis cohort showed significantly higher IgG responses to *B. pseudomallei* (median of optical density [OD] = 1.42), *B. thailandensis* (median OD = 1.12), and BTCV (median OD = 1.43) than any of the control cohorts (p<0.01) (Figure 2, panel A). IgM responses to *B. pseudomallei* (median OD = 0.48) in the melioidosis cohort, similar to IgG responses, were higher than the control cohorts (median OD ranges 0.19–0.28, p<0.02). In contrast, IgM responses to *B. thailandensis* and BTCV in the melioidosis cohort were similar to responses in the HH cohort but higher than those in DM and OGNI (p<0.05) (Figure 2, panel B). In the melioidosis cohort, we observed a strong correlation from both IgM and IgG responses between *B. pseudomallei*, *B. thailandensis*, and BTCV (ρ>0.9; p<0.01) (Appendix Tables 2, 3).

### Interferon-γ ELISpot Responses to *B. pseudomallei*, *B. thailandensis*,and BTCV

**Figure 3:**
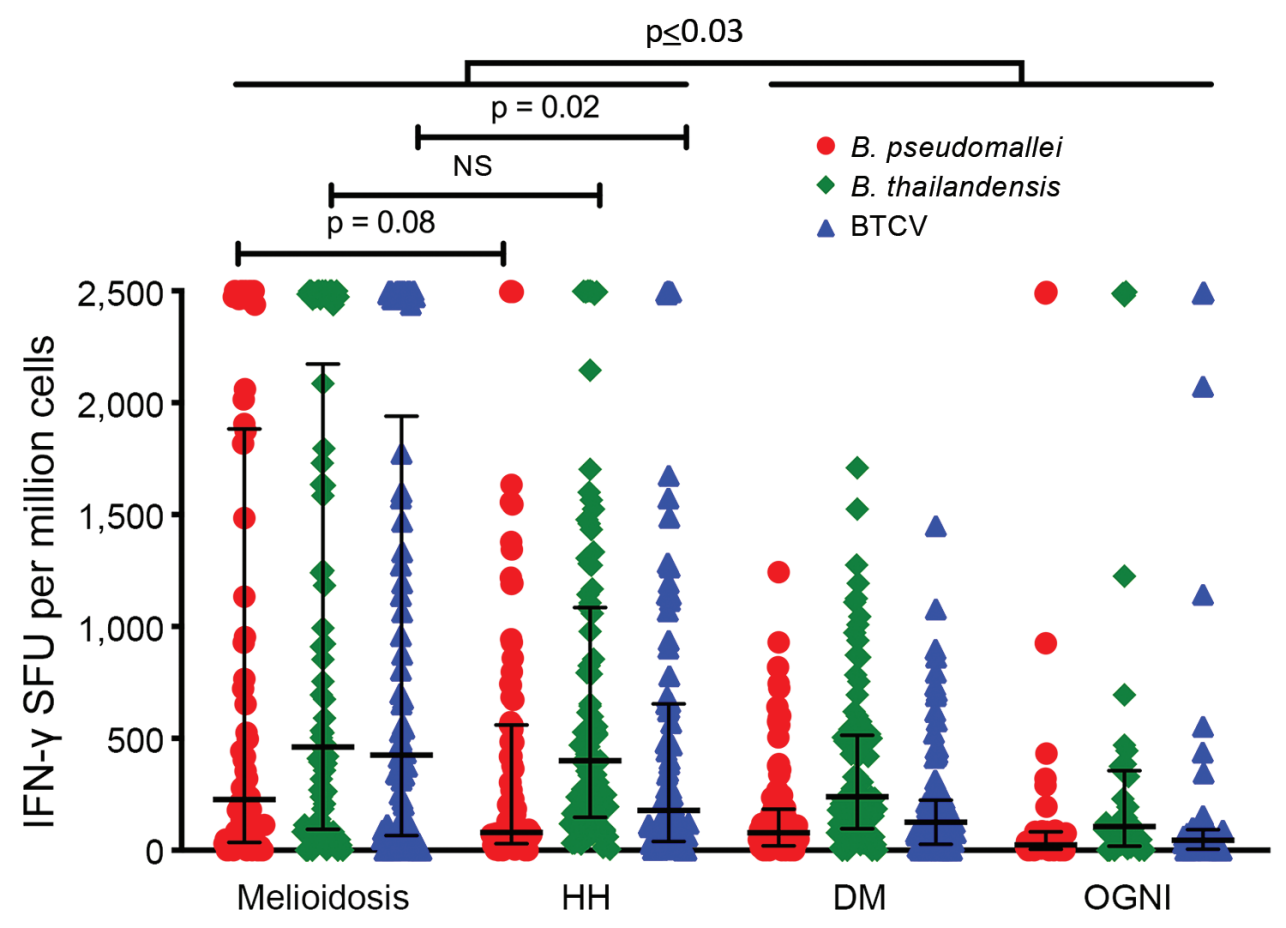
Ex vivo IFN-γ ELISpot responses to *Burkholderia pseudomallei*, *B. thailandensis*, and BTCV, Thailand. IFN-γ responses were quantified for acute melioidosis patients (n = 82) and 3 control cohorts: HH, n = 93), diabetic patients (DM, n = 95), and patients with other gram-negative infections (OGNI, n = 42) against whole-cell heat-killed antigens of *Burkholderia pseudomallei* (BP, red dots), *Burkholderia thailandensis* (BT, green diamonds), and *Burkholderia thailandensis CPS variant* (BTCV, blue triangles) are shown. Each symbol represents the average number of SFU per subject. Medians (horizontal lines) and interquartile ranges (error bars) are shown. p values were calculated by using the nonparametric Mann-Whitney test. Horizontal bars at top of figure indicate comparisons across cohorts. BTCV, *B. thailandensis* CPS variant; CPS, capsular polysaccharide; DM, patients with diabetes mellitus; HH, healthy household contacts of melioidosis case-patients; IFN-γ, interferon-γ; NS, not significant; SFU, spot-forming units.

Quantitatively, IFN-γ responses against *B. pseudomallei* in the melioidosis and HH cohorts were significantly higher than those for the DM and OGNI cohorts (p<0.03). We observed similar outcomes in the responses to *B. thailandensis* and BTCV (Figure 3). Of interest, the melioidosis and HH cohorts showed comparable IFN-γ responses against *B. pseudomallei* and *B. thailandensis*, but not to BTCV (p = 0.02) (Figure 3). In the melioidosis cohort, we observed strong correlations between IFN-γ responses to *B. pseudomallei*, *B. thailandensis*, and BTCV (ρ>0.9; p<0.01). We observed similar correlations (ρ>0.7; p<0.01) for the HH, OGNI, and DM cohorts (Appendix Table 4).

### Cellular Immune Responses to *B. pseudomallei*, *B. thailandensis*, and BTCV by Whole-Blood Stimulation Assay Using Flow Cytometry

**Figure 4:**
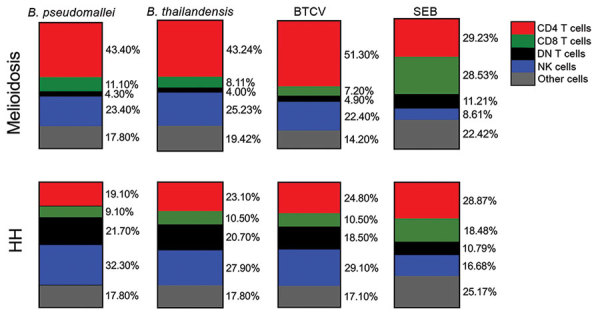
Cellular immune responses to *Burkholderia pseudomallei*, *B. thailandensis*, and BTCV by whole-blood stimulation assay using flow cytometry between melioidosis patients and healthy persons in *B. pseudomallei*–endemic areas, Thailand. Whole blood samples from 14 patients with acute melioidosis and 8 HH contacts were stimulated with culture-filtrate antigens of *B. pseudomallei*, *B. thailandensis*, BTCV, and SEB (positive control). Frequencies of CD4, CD8, and DN T cells; NK cells; and other cells within total IFN-γ–producing cells are shown. Medians were used to generate each vertical slice graph. BTCV, *B. thailandensis* CPS variant; CPS, capsular polysaccharide; DM, patients with diabetes mellitus; DN, double negative (CD4^–^CD8^–^); HH, healthy household contacts of melioidosis case-patients; IFN-γ, interferon-γ; SEB, *Staphylococcus* enterotoxin B.

To determine the contribution of CD4 T, CD8 T, DN T, and NK cells to total IFN-γ responses in the melioidosis and HH cohorts, we performed multicolor flow cytometry on WBA samples. In the melioidosis cohort, about half of the IFN-γ responses, on average, came from CD4 T cells for all 3 *Burkholderia* antigens (Figure 4), which was significantly higher than for the HH cohort (p<0.03), suggesting a strong contribution of antigen specific memory responses (Appendix Figure 1, panels A, E). In contrast, the IFN-γ responses in the HH cohort were primarily driven by NK cells (about one third), followed by a balanced mix of DN T, CD4 T, and other cells (Figure 4). In particular, the contribution of IFN-γ responses from DN T cells against all *Burkholderia* antigens in the HH cohort was significantly higher than in the melioidosis cohort (p<0.01) (Appendix Figure 1, panel C). In the melioidosis cohort, we observed a strong correlation of IFN-γ responses against *B. pseudomallei*, *B. thailandensis*, and BTCV in CD4 T cells (ρ>0.8; p<0.01) (Appendix Table 5). In contrast, the HH cohort showed strong correlation between IFN-γ responses toward *B. pseudomallei*, *B. thailandensis*, and BTCV in CD8 T and NK cells (ρ>0.9; p<0.01) (Appendix Table 6).

## Discussion

Our results demonstrate that melioidosis patients show strong humoral and cellular cross-immunity between the pathogenic *B. pseudomallei* and the less pathogenic *B. thailandensis* and BTCV. On the other hand, the HH cohort and control cohorts in general had relatively low humoral immune responses to all 3 *Burkholderia* antigens but profound cellular immune responses. We showed that just over half of the acute melioidosis patients were positive by IHA; previous work in our group has shown that a significant proportion (12.6%) of melioidosis patients never seroconvert (*16*). We do not have enough information to confirm whether the persistence of seropositivity against *B. pseudomallei* in healthy persons is associated with latent infection or with successful clearance of *B. pseudomallei* after exposure events.

Seropositivity against *B. pseudomallei* is associated with repeated environmental exposure to the organism (*19*). In addition, a recent epidemiologic study showed high prevalence of environmental *B. pseudomallei* in rice paddy fields across multiple regions of Thailand, especially in the east (*6*). Subsequent serologic responses against *B. pseudomallei* in healthy rice farmers are associated with exposure to environmental *B. pseudomallei* rather than *B. thailandensis* and BTCV (*20*); the cause could be a higher prevalence of *B. pseudomallei*. It is impossible to distinguish between the serologic responses to *B. pseudomallei* and BTCV because CPS components are highly cross-reactive (*9*).

Humoral immune responses against CPS components have been found to be associated with protection against experimental melioidosis in mice (*22*,*23*). In a previous study, Tiyawisutsri et al. used modified IHA to detect cross-reactivity between *B. pseudomallei*, *B. thailandensis*, and *B. mallei*, the causative agent of glanders, in melioidosis patients (*24*). Tiyawisutsri et al. reported poor cross-reactivity between *B. pseudomallei* and *B. thailandensis* and saw cross-reactivity to *B. mallei*, which expresses similar CPS components (*24*,*25*). Consistent with these findings, we report strong cross-reactivity between *B. pseudomallei* and BTCV, and to a lesser extent to *B. thailandensis* in the melioidosis and HH cohorts. Moreover, we suggest that the IHA responses may be primarily specific to CPS components that are not present in *B. thailandensis* (*9*). We explored the humoral responses by IgM and IgG; we report strong IgG responses in acute melioidosis patients but marginal IgM responses. A possible cause of the low IgM responses in acute melioidosis patients is that disease onset occurred some time before admission; reported incubation period is 1–21 days (*26*). Another possibility is that preexisting immunity to *B. pseudomallei* resulted in a burst of IgG responses around the time of study enrollment, which is consistent with a previous report (*27*). Thus, the IgG ELISA could be an improved diagnostic method for melioidosis (*28*).

Cell-mediated immune responses by IFN-γ and type I immune responses (e.g., interleukin [IL] 12, IL-18, tumor necrosis factor α) are essential for the host immune system in fighting against intracellular infections (*29**–**31*). Nithichanon et al. showed that most of an exposed healthy population has acquired cellular immunity against broad immunogenic *B. pseudomallei* epitopes (*32*). Our IFN-γ ELISpot experiments suggest that both melioidosis patients and HH contacts engage strong cross-immune IFN-γ responses between *B. pseudomallei*, *B. thailandensis*, and BTCV, despite low humoral responses in the HH cohort. The IFN-γ responses predominantly from T cells during melioidosis are associated with protection and survival against *B. pseudomallei* infection (*13*). We demonstrate that IFN-γ responses to all 3 antigens are a mix of T and NK cell responses, with different contribution of T-cell subsets in the melioidosis cohort compared with the HH cohort. Whereas melioidosis patients predominantly exhibit CD4 T-cell IFN-γ responses, the HH cohort is characterized by a mix of double negative and CD4 T-cell responses and increased NK cell responses, suggesting an innate or innate-like driven immune response.

Low-dose exposure to *B. pseudomallei* in healthy persons may also contribute to immune responses. *B. pseudomallei*–specific cellular immunity in seronegative healthy participants showed detectable IFN-γ ELISpot responses in some subjects to virulent factors in *B. pseudomallei*, such as bopE (Type III secreted protein), pilO (Type IV pilus biosynthesis protein), and flgK (flagellar hook-associated protein) (*12*). Another possibility would be cross-reactivity to other gram-negative bacteria, as demonstrated by some OGNI participants exhibiting high IFN-γ responses to whole-cell heat-killed *Burkholderia* antigens (Figure 3).

In a previous study in this population, we demonstrated that acute melioidosis patients elicited strong cellular immune responses in both the CD4 and CD8 T-cell compartments (*13*). Cellular immune responses by CD4 T cells against *B. pseudomallei* antigen (AhpC) have been associated with survival (*33*). HIV positivity does not seem to be a major risk factor for melioidosis; a surge in melioidosis incidence and severity was not seen during the HIV epidemic in the 1990s in Thailand (*34*) although cases of tuberculosis and other opportunistic infections such as cryptococcal meningitis did increase. During this time, 2.8% (95% CI 0.8%–4.7%) of 286 patients in Ubon Ratchathani with *B. pseudomallei* tested positive for HIV, compared with 0.6%–1.1% of blood donors, but this difference was not significant (*35*). However, HIV infection is known to increase the risk for other gram-negative infections, such as *Salmonella* and *Escherichia coli*, and it is likely that a relationship between HIV and melioidosis could be demonstrated by sufficiently powered studies. To date, host immune responses by CD4 T cells in HIV-positive persons with melioidosis remain unknown. Host immune responses during acute melioidosis associated with survival have been shown to be dominated by CD8 T and NK cells (*36*,*37*), and some redundancy may occur to allow compensation of low CD4 counts in HIV.

In conclusion, patients with melioidosis in Thailand demonstrate immune cross-reactivity between *B. pseudomallei*, *B. thailandensis*, and BTCV in both humoral and cellular immune compartments. Healthy persons who live in melioidosis-endemic areas, on the other hand, primarily demonstrate cellular immune cross-reactivity. We recommend further investigation of human immune responses in healthy persons where the less pathogenic strains are prevalent, such as in the central and eastern parts of Thailand (*20*). It is possible that exposure to the less virulent *B. thailandensis* and BTCV generates immune cross-reactivity, which could confer some protection against melioidosis. Nevertheless, cross-protection against *B. pseudomallei* infection through immune cross-reactivity in humans requires further study. Understanding the consequences of naturally acquired immunity to *B. pseudomallei* or *B. thailandensis* variants in previously exposed populations is particularly needed for the development of an efficacious vaccine.

Appendix Additional information about human immune responses to melioidosis and cross-reactivity to low-virulence *Burkholderia* species, Thailand.

## References

[1] Limmathurotsakul D, Golding N, Dance DAB, Messina JP, Pigott DM, Moyes CL, et al. Predicted global distribution of *Burkholderia pseudomallei* and burden of melioidosis. Nat Microbiol. 2016;1:15008. 10.1038/nmicrobiol.2015.8 27571754

[2] Currie BJ, Dance DA, Cheng AC. The global distribution of *Burkholderia pseudomallei* and melioidosis: an update. Trans R Soc Trop Med Hyg. 2008;102(Suppl 1):S1–4. 10.1016/S0035-9203(08)70002-6 19121666

[3] Stewart JD, Smith S, Binotto E, McBride WJ, Currie BJ, Hanson J. The epidemiology and clinical features of melioidosis in Far North Queensland: Implications for patient management. PLoS Negl Trop Dis. 2017;11:e0005411. 10.1371/journal.pntd.0005411 28264029 PMC5363997

[4] Zheng Y, Ley SH, Hu FB. Global aetiology and epidemiology of type 2 diabetes mellitus and its complications. Nat Rev Endocrinol. 2018;14:88–98. 10.1038/nrendo.2017.151 29219149

[5] Trakulsomboon S, Vuddhakul V, Tharavichitkul P, Na-Gnam N, Suputtamongkol Y, Thamlikitkul V. Epidemiology of arabinose assimilation in *burkholderia pseudomallei* isolated from patients and soil in Thailand. Southeast Asian J Trop Med Public Health. 1999;30:756–9. 10928371

[6] Hantrakun V, Rongkard P, Oyuchua M, Amornchai P, Lim C, Wuthiekanun V, et al. Soil nutrient depletion is associated with the presence of *Burkholderia pseudomallei.* Appl Environ Microbiol. 2016;82:7086–92. 10.1128/AEM.02538-16 27694236 PMC5118919

[7] Kim HS, Schell MA, Yu Y, Ulrich RL, Sarria SH, Nierman WC, et al. Bacterial genome adaptation to niches: divergence of the potential virulence genes in three *Burkholderia* species of different survival strategies. BMC Genomics. 2005;6:174. 10.1186/1471-2164-6-174 16336651 PMC1343551

[8] Moore RA, Reckseidler-Zenteno S, Kim H, Nierman W, Yu Y, Tuanyok A, et al. Contribution of gene loss to the pathogenic evolution of *Burkholderia pseudomallei* and *Burkholderia mallei.* Infect Immun. 2004;72:4172–87. 10.1128/IAI.72.7.4172-4187.2004 15213162 PMC427422

[9] Sim BM, Chantratita N, Ooi WF, Nandi T, Tewhey R, Wuthiekanun V, et al. Genomic acquisition of a capsular polysaccharide virulence cluster by non-pathogenic *Burkholderia* isolates. Genome Biol. 2010;11:R89. 10.1186/gb-2010-11-8-r89 20799932 PMC2945791

[10] Scott AE, Laws TR, D’Elia RV, Stokes MG, Nandi T, Williamson ED, et al. Protection against experimental melioidosis following immunization with live *Burkholderia thailandensis* expressing a *manno*-heptose capsule. Clin Vaccine Immunol. 2013;20:1041–7. 10.1128/CVI.00113-13 23677322 PMC3697456

[11] Tippayawat P, Saenwongsa W, Mahawantung J, Suwannasaen D, Chetchotisakd P, Limmathurotsakul D, et al. Phenotypic and functional characterization of human memory T cell responses to *Burkholderia pseudomallei.* PLoS Negl Trop Dis. 2009;3:e407. 10.1371/journal.pntd.0000407 19352426 PMC2660609

[12] Dunachie SJ, Jenjaroen K, Reynolds CJ, Quigley KJ, Sergeant R, Sumonwiriya M, et al. Infection with *Burkholderia pseudomallei* - immune correlates of survival in acute melioidosis. Sci Rep. 2017;7:12143. 10.1038/s41598-017-12331-5 28939855 PMC5610189

[13] Jenjaroen K, Chumseng S, Sumonwiriya M, Ariyaprasert P, Chantratita N, Sunyakumthorn P, et al. T-cell responses are associated with survival in acute melioidosis patients. PLoS Negl Trop Dis. 2015;9:e0004152. 10.1371/journal.pntd.0004152 26495852 PMC4619742

[14] Ketheesan N, Barnes JL, Ulett GC, VanGessel HJ, Norton RE, Hirst RG, et al. Demonstration of a cell-mediated immune response in melioidosis. J Infect Dis. 2002;186:286–9. 10.1086/341222 12134268

[15] Healey GD, Elvin SJ, Morton M, Williamson ED. Humoral and cell-mediated adaptive immune responses are required for protection against *Burkholderia pseudomallei* challenge and bacterial clearance postinfection. Infect Immun. 2005;73:5945–51. 10.1128/IAI.73.9.5945-5951.2005 16113315 PMC1231116

[16] Chaichana P, Jenjaroen K, Amornchai P, Chumseng S, Langla S, Rongkard P, et al. Antibodies in melioidosis: the role of the indirect hemagglutination assay in evaluating patients and exposed populations. Am J Trop Med Hyg. 2018;99:1378–85. 10.4269/ajtmh.17-0998 30298810 PMC6283516

[17] Alexander AD, Huxsoll DL, Warner AR Jr, Shepler V, Dorsey A. Serological diagnosis of human melioidosis with indirect hemagglutination and complement fixation tests. Appl Microbiol. 1970;20:825–33. 10.1128/AEM.20.5.825-833.1970 5530276 PMC377056

[18] Chantratita N, Wuthiekanun V, Thanwisai A, Limmathurotsakul D, Cheng AC, Chierakul W, et al. Accuracy of enzyme-linked immunosorbent assay using crude and purified antigens for serodiagnosis of melioidosis. Clin Vaccine Immunol. 2007;14:110–3. 10.1128/CVI.00289-06 17093104 PMC1797717

[19] Wuthiekanun V, Chierakul W, Langa S, Chaowagul W, Panpitpat C, Saipan P, et al. Development of antibodies to *Burkholderia pseudomallei* during childhood in melioidosis-endemic northeast Thailand. Am J Trop Med Hyg. 2006;74:1074–5. 10.4269/ajtmh.2006.74.1074 16760522

[20] Hantrakun V, Thaipadungpanit J, Rongkard P, Srilohasin P, Amornchai P, Langla S, et al. Presence of *B. thailandensis* and *B. thailandensis* expressing *B. pseudomallei*-like capsular polysaccharide in Thailand, and their associations with serological response to *B. pseudomallei.* PLoS Negl Trop Dis. 2018;12:e0006193. 10.1371/journal.pntd.0006193 29364892 PMC5809093

[21] Suttisunhakul V, Chantratita N, Wikraiphat C, Wuthiekanun V, Douglas Z, Day NPJ, et al. Evaluation of polysaccharide-based latex agglutination assays for the rapid detection of antibodies to *Burkholderia pseudomallei.* Am J Trop Med Hyg. 2015;93:542–6. 10.4269/ajtmh.15-0114 26123956 PMC4559694

[22] Burtnick MN, Shaffer TL, Ross BN, Muruato LA, Sbrana E, DeShazer D, et al. Development of subunit vaccines that provide high-level protection and sterilizing immunity against acute inhalational melioidosis. Infect Immun. 2017;86:e00724–17. 10.1128/IAI.00724-17 29109172 PMC5736816

[23] Scott AE, Burtnick MN, Stokes MG, Whelan AO, Williamson ED, Atkins TP, et al. *Burkholderia pseudomallei* capsular polysaccharide conjugates provide protection against acute melioidosis. Infect Immun. 2014;82:3206–13. 10.1128/IAI.01847-14 24866807 PMC4136211

[24] Tiyawisutsri R, Peacock SJ, Langa S, Limmathurotsakul D, Cheng AC, Chierakul W, et al. Antibodies from patients with melioidosis recognize *Burkholderia mallei* but not *Burkholderia thailandensis* antigens in the indirect hemagglutination assay. J Clin Microbiol. 2005;43:4872–4. 10.1128/JCM.43.9.4872-4874.2005 16145163 PMC1234129

[25] Heiss C, Burtnick MN, Wang Z, Azadi P, Brett PJ. Structural analysis of capsular polysaccharides expressed by *Burkholderia mallei* and *Burkholderia pseudomallei.* Carbohydr Res. 2012;349:90–4. 10.1016/j.carres.2011.12.011 22221792

[26] Currie BJ, Fisher DA, Howard DM, Burrow JN, Selvanayagam S, Snelling PL, et al. The epidemiology of melioidosis in Australia and Papua New Guinea. Acta Trop. 2000;74:121–7. 10.1016/S0001-706X(99)00060-1 10674639

[27] Currie BJ, Fisher DA, Anstey NM, Jacups SP. Melioidosis: acute and chronic disease, relapse and re-activation. Trans R Soc Trop Med Hyg. 2000;94:301–4. 10.1016/S0035-9203(00)90333-X 10975006

[28] Suttisunhakul V, Wuthiekanun V, Brett PJ, Khusmith S, Day NP, Burtnick MN, et al. Development of rapid enzyme-linked immunosorbent assays for detection of antibodies to *Burkholderia pseudomallei.* J Clin Microbiol. 2016;54:1259–68. 10.1128/JCM.02856-15 26912754 PMC4844749

[29] Trinchieri G. Cytokines acting on or secreted by macrophages during intracellular infection (IL-10, IL-12, IFN-gamma). Curr Opin Immunol. 1997;9:17–23. 10.1016/S0952-7915(97)80154-9 9039773

[30] Dinarello CA, Fantuzzi G. Interleukin-18 and host defense against infection. J Infect Dis. 2003;187(Suppl 2):S370–84. 10.1086/374751 12792854

[31] Barnes JL, Williams NL, Ketheesan N. Susceptibility to *Burkholderia pseudomallei* is associated with host immune responses involving tumor necrosis factor receptor-1 (TNFR1) and TNF receptor-2 (TNFR2). FEMS Immunol Med Microbiol. 2008;52:379–88. 10.1111/j.1574-695X.2008.00389.x 18294191

[32] Nithichanon A, Rinchai D, Buddhisa S, Saenmuang P, Kewcharoenwong C, Kessler B, et al. Immune control of *Burkholderia pseudomallei*—common, high-frequency T-cell responses to a broad repertoire of immunoprevalent epitopes. Front Immunol. 2018;9:484. 10.3389/fimmu.2018.00484 29616023 PMC5869189

[33] Reynolds C, Goudet A, Jenjaroen K, Sumonwiriya M, Rinchai D, Musson J, et al. T cell Immunity to the alkyl hydroperoxide reductase of *Burkholderia pseudomallei:* a correlate of disease outcome in acute melioidosis. J Immunol. 2015;194:4814–24. 10.4049/jimmunol.1402862 25862821 PMC4416739

[34] Chierakul W, Wuthiekanun V, Chaowagul W, Amornchai P, Cheng AC, White NJ, et al. Short report: disease severity and outcome of melioidosis in HIV coinfected individuals. Am J Trop Med Hyg. 2005;73:1165–6. 10.4269/ajtmh.2005.73.1165 16354832

[35] Chierakul W, Rajanuwong A, Wuthiekanun V, Teerawattanasook N, Gasiprong M, Simpson A, et al. The changing pattern of bloodstream infections associated with the rise in HIV prevalence in northeastern Thailand. Trans R Soc Trop Med Hyg. 2004;98:678–86. 10.1016/j.trstmh.2004.01.011 15363648

[36] Kronsteiner B, Chaichana P, Sumonwiriya M, Jenjaroen K, Chowdhury FR, Chumseng S, et al. Diabetes alters immune response patterns to acute melioidosis in humans. Eur J Immunol. 2019;49:1092–106. 10.1002/eji.201848037 31032897 PMC6618312

[37] Lertmemongkolchai G, Cai G, Hunter CA, Bancroft GJ. Bystander activation of CD8+ T cells contributes to the rapid production of IFN-gamma in response to bacterial pathogens. J Immunol. 2001;166:1097–105. 10.4049/jimmunol.166.2.1097 11145690

