# Supplementary material for: Human Immune Responses to Melioidosis and Cross-Reactivity to Low-Virulence Burkholderia Species, Thailand
Source: Emerg Infect Dis. 2020 Mar;26(3):463–71. doi: 10.3201/eid2603.190206 (PMC7045851; doi:10.3201/eid2603.190206)
Supplement: Appendix — Additional information about human immune responses to melioidosis and cross-reactivity to low-virulence Burkholderia species, Thailand. [file 19-0206-Techapp-s1.pdf]

Article DOI: https://doi.org/10.3201/eid2603.190206

Human Immune Responses to Melioidosis and Cross-Reactivity to Low-Virulence *Burkholderia* Species, Thailand

Appendix

Appendix Table 1. Correlation coefficients (Spearman's ρ) for indirect hemagglutination assays against 3 *Burkholderia* antigens in 4 study cohorts*

	Melioidosis (n = 73)		HH (n = 35)		DM (n = 54)		OGNI (n = 10)	
Antigen	BP	BT	BP	BT	BP	BT	BP	BT
Burkholderia pseudomallei								
Burkholderia thailandensis	0.53†		0.19‡		0.27‡		0.05‡	
Burkholderia thailandensis CPS variant	0.96†	0.59†	0.84†	0.07‡	0.88†	0.25‡	0.67‡	0.58‡

*BP, *Burkholderia pseudomallei*; BT, *Burkholderia thailandensis*; BTCV, *Burkholderia thailandensis* CPS variant; DM, study participants attending the hospital's diabetes outpatient clinic; HH, healthy household contacts of the melioidosis patients; OGNI, hospital patients with other gram-negative bacterial infections.
†Correlation is significant at the <0.05 level (2-tailed).
‡Correlation is not significant.

Appendix Table 2. Correlation coefficients (Spearman's ρ) for IgM ELISA against 3 *Burkholderia* antigens in 4 study cohorts*

	Melioidosis (n = 73)		HH (n = 35)		DM (n = 54)		OGNI (n = 10)	
Antigen	BP	BT	BP	BT	BP	BT	BP	BT
Burkholderia pseudomallei								
Burkholderia thailandensis	0.95		0.92		0.87		0.98	
Burkholderia thailandensis CPS variant	0.96	0.94	0.93	0.93	0.87	0.98	0.96	0.98

*Correlation is significant at the ≤0.01 level (2-tailed). BP, *Burkholderia pseudomallei*; BT, *Burkholderia thailandensis*; BTCV, *Burkholderia thailandensis* CPS variant. DM, study participants attending the hospital's diabetes outpatient clinic; HH, healthy household contacts of the melioidosis patients; OGNI, hospital patients with other gram-negative bacterial infections.

Appendix Table 3. Correlation coefficients (Spearman's ρ) for IgG ELISA 3 *Burkholderia* antigens in 4 study cohorts*

	Melioidosis (n = 73)		HH (n = 35)		DM (n = 54)		OGNI (n = 10)	
Antigen	BP	BT	BP	BT	BP	BT	BP	BT
Burkholderia pseudomallei								
Burkholderia thailandensis	0.95		0.93		0.92		0.89	
Burkholderia thailandensis CPS variant	0.97	0.93	0.97	0.94	0.35	0.42	0.95	0.98

*Correlation is significant at the ≤0.01 level (2-tailed). BP, *Burkholderia pseudomallei*; BT, *Burkholderia thailandensis;* BTCV, *Burkholderia thailandensis* CPS variant; DM, study participants attending the hospital's diabetes outpatient clinic; HH, healthy household contacts of the melioidosis patients; OGNI, hospital patients with other gram-negative bacterial infections.

Appendix Table 4. Correlation coefficients (Spearman's ρ) for interferon-gamma enzyme-linked immunospot assay against 3 *Burkholderia* antigens in 4 study cohorts*

	Melioidosis (n = 82)		HH (n = 93)		DM (n = 95)		OGNI (n = 42)	
Antigen	BP	BT	BP	BT	BP	BT	BP	BT
Burkholderia pseudomallei								
Burkholderia thailandensis	0.94		0.87		0.74		0.80	
Burkholderia thailandensis CPS variant	0.95	0.98	0.95	0.88	0.93	0.78	0.85	0.85

*Correlation is significant at the ≤0.01 level (2-tailed). BP, *Burkholderia pseudomallei*; BT, *Burkholderia thailandensis;* BTCV, *Burkholderia thailandensis* CPS variant; DM, study participants attending the hospital's diabetes outpatient clinic; HH, healthy household contacts of the melioidosis patients; OGNI, hospital patients with other gram-negative bacterial infections.

Appendix Table 5. Correlation coefficients among interferon-gamma–producing CD4 T cells from whole-blood assay against 3 *Burkholderia* antigens in patients with melioidosis*

	CD4		CD8		DN T		NK	
Antigen	BP	BT	BP	BT	BP	BT	BP	BT
Burkholderia pseudomallei								
Burkholderia thailandensis	0.89†		0.33‡		–0.11‡		0.70†	
Burkholderia thailandensis CPS variant	0.81†	0.88†	0.52‡	0.67†	0.66†	–0.01‡	0.75†	0.95†

*BP, *Burkholderia pseudomallei*; BT, *Burkholderia thailandensis;* BTCV, *Burkholderia thailandensis CPS variant;* CD4, CD4 T cell; CD8, CD8 T cell; DN T, double negative T cell; NK, natural killer cell.
†Correlation is significant at the ≤0.01 level (2-tailed).
‡Correlation is not significant.

Appendix Table 6. Correlation coefficients among interferon-gamma–producing CD4 T cells from whole-blood assay of *Burkholderia* antigens in healthy household contacts of patients with melioidosis

	CD4		CD8		DN T		NK	
Antigen	BP	BT	BP	BT	BP	BT	BP	BT
Burkholderia pseudomallei								
Burkholderia thailandensis	0.88†		0.95†		0.95†		0.91†	
Burkholderia thailandensis CPS variant	0.69‡	0.71‡	0.98†	0.98†	0.83†	0.71‡	0.95†	0.91†

*BP, *Burkholderia pseudomallei*; BT, *Burkholderia thailandensis;* BTCV, *Burkholderia thailandensis CPS variant;* CD4, CD4 T cell; CD8, CD8 T cell; DN T, double negative T cell; NK, natural killer cell.
†Correlation is significant at the ≤0.01 level (2-tailed).
‡Correlation is not significant;

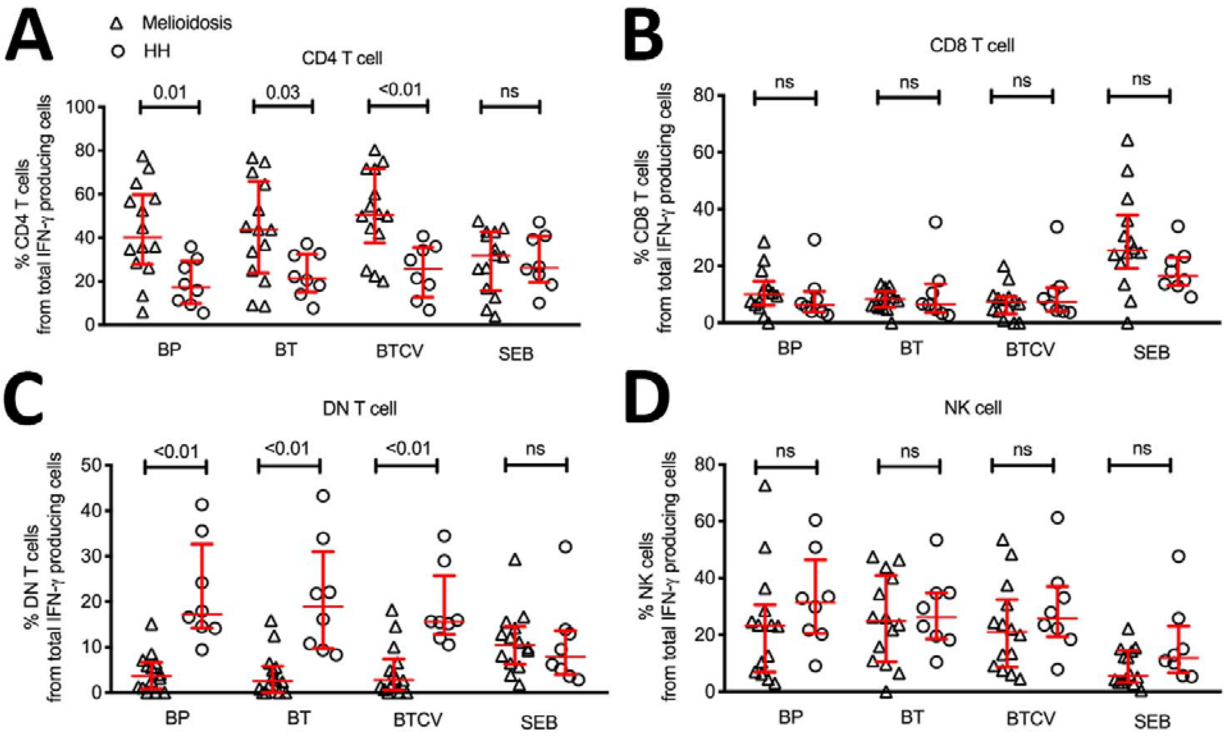

Appendix Figure. Cellular immune responses to *Burkholderia pseudomallei*, *Burkholderia thailandensis,* and *Burkholderia thailandensis* CPS variant by whole-blood stimulation assay using flow cytometry between melioidosis patients and healthy persons in BP-endemic areas. Whole blood samples from 14 melioidosis patients (Melioidosis, triangle) and 8 healthy household contacts of the melioidosis patients (HH, circle) were stimulated with culture-filtrate antigens of *Burkholderia pseudomallei* (BP), *Burkholderia thailandensis* (BT), *Burkholderia thailandensis* CPS variant (BTCV) and *Staphylococcus* enterotoxin B (SEB, positive control). Frequencies of CD4, CD8, $CD4^- CD8^-$ (double negative, DN) T-cells, and NK cells from total interferon-gamma γ (IFN-γ, A-D) and tumor necrosis factor α (TNF-α) producing cells are shown. Each symbol represents the cellular immune response from an individual. Medians and interquartile ranges are shown in the graphs. p values were calculated from non-parametric Mann-Whitney test and indicated on the plots; ns, not significant.
